# Cardiac arrhythmias in patients with SARS-CoV‑2 infection and effects of the lockdown on invasive rhythmological therapy

**DOI:** 10.1007/s00399-020-00734-3

**Published:** 2020-12-23

**Authors:** Shibu Mathew, Christian Fraebel, Victoria Johnson, Saboukh Abdelgwad, Nikita Schneider, Patrick Müller, Ritvan Chasan, Christian Hamm, Joern Schmitt

**Affiliations:** 1 Med. Klinik I—Department of cardiology, University Hospital Giessen, Klinikstraße 33, 35390 Giessen, Germany; 2 Department of cardiology, University Hospital Muenster, Muenster, Germany

**Keywords:** COVID-19, Catheter ablation, Pacemaker, Electrophysiology, Pandemic, COVID-19, Katheterablation, Herzschrittmacher, Elektrophysiologie, Pandemie

## Abstract

**Background:**

Since the outbreak of the coronavirus disease 2019 (COVID-19) pandemic, various strategies have been taken worldwide to reduce the risk of infection. As part of the amendment to the Infection Protection Act, elective medical interventions were restricted, leading to a change in patient care. However, the consequences of the lockdown on the treatment of rhythmological patients in Germany remains unclear.

**Objectives:**

The aim of this study was to analyze the reduction in rhythmological interventions and the patient care situation using a nationwide survey during the first lockdown period.

**Methods:**

A survey was sent to all electrophysiological centers certified by the German Society of Cardiology. Here, the treatment volume of tachycardia and bradycardia and their invasive therapy were surveyed before and during the lockdown period. Furthermore, the number of patients with severe acute respiratory syndrome coronavirus 2 (SARS-CoV-2) treated at these centers and the incidence of cardiac arrhythmias was also recorded.

**Results:**

Participating centers performed a total of 24,648 ablation procedures/year and represent approximately 34% (24,648/72,548) of the estimated German ablation treatments. The majority of these centers (33/40; 82.5%) were so-called primary COVID-19 hospitals (level-1). Overall, the number of ablations and pacemaker implantations were reduced by 41% and 18% respectively. Due to postponed ablation procedures and pacemaker implantations, 22/40 (55%) centers reported a worsening of clinical symptoms or early re-hospitalization of their patients.

**Conclusion:**

These results demonstrate a significant decline in elective rhythmological procedures during the lockdown, as required by the German Federal Government. At the same time, however, more than half of the participating centers reported an increase in patient re-hospitalizations due to postponed procedures.

## Abstract (de)

**Hintergrund:**

Seit Ausbruch der Coronavirus-disease-19(COVID-19)-Pandemie wurden zur Eindämmung der Infektionsgefahr weltweit vielfältige Maßnahmen ergriffen. Im Rahmen der Anpassung des Infektionsschutzgesetzes und der damit verbundenen Restriktion elektiver Eingriffe ist von deutlichen Veränderungen in der Patientenversorgung auszugehen. Jedoch ist unklar, welchen Einfluss der erste Lockdown in Deutschland auf die Versorgungssituation rhythmologischer Patienten hatte.

**Zielsetzung:**

Ziel dieser Arbeit war es, anhand einer bundesweiten Umfrage die tatsächliche Reduktion rhythmologischer Eingriffe sowie die Versorgungssituation im ersten Lockdown zu analysieren.

**Methodik:**

Ein Fragebogen wurde an alle von der Deutschen Gesellschaft für Kardiologie zertifizierten elektrophysiologischen Zentren versendet. Hier wurden das Behandlungsvolumen tachykarder und bradykarder Herzrhythmusstörungen und deren invasive Therapie vor und im Lockdown-Zeitraum abgefragt. Ferner wurden die Anzahl der behandelten Patienten mit dem „severe acute respiratory syndrome coronavirus 2“ (SARS-CoV-2) in diesen Zentren sowie die Inzidenz einzelner Herzrhythmusstörungen erfasst.

**Ergebnisse:**

Die teilnehmenden Zentren führten insgesamt 24.648 Ablationseingriffe pro Jahr durch und decken etwa 34 % (24.648/72.548) der geschätzten deutschen Ablationsbehandlungen ab. Bei diesen handelte es sich zu einem Großteil (33/40; 82,5 %) um sogenannte primäre COVID-19-Krankenhäuser (Level 1). Insgesamt kam es zu einer Reduktion der Zahl an Ablationen und Herzschrittmacherimplantationen um 41 % und 18 %. Insgesamt 22/40 (55 %) Zentren gaben an, aufgrund verschobener Ablationen oder Herzschrittmacherimplantationen eine Verschlechterung der klinischen Symptomatik oder eine vorzeitige Rehospitalisierung ihrer Patienten beobachtet zu haben.

**Schlussfolgerung:**

Diese Ergebnisse demonstrieren, wie von der Politik gefordert, einen deutlichen Rückgang der Elektivprozeduren während des initialen Lockdowns im Bereich der Rhythmologie. Zeitgleich berichtete jedoch mehr als die Hälfte der teilnehmenden Zentren über eine Zunahme an Rehospitalisierungen der Patienten aufgrund der verschobenen Eingriffe.

## Introduction

Since the outbreak of coronavirus disease 19 (COVID-19) in Wuhan, China, in December 2019, the pandemic has rapidly spread worldwide. On March 12th, 2020, the World Health Organization (WHO) declared this global outbreak of severe acute respiratory syndrome coronavirus 2 (SARS-CoV-2) to be a pandemic. At the beginning of April, 1.5 million people worldwide were affected by SARS-CoV‑2, according to John Hopkins University. At this point, approximately 90,000 people had died as a result of the disease [1]. SARS-CoV‑2 is a primary respiratory viral infection. The main clinical symptoms include loss of taste, fever, and cough [2, 3]. It can currently be assumed that the majority of patients have a mild course (81%), but 14% can be severely affected and up to 5% become critically ill [2]. The virus was found to be highly contagious, so a wide range of measures have been taken worldwide to limit the spread of the virus.

In this context, on March 13th, 2020, the Federal Government of Germany asked all hospitals to postpone all elective procedures that were not essential from a medical point of view in order to create additional intensive care capacities. In addition, a lockdown was imposed on the population of the Federal Republic of Germany to slow down the spread of the virus. Due to a decreased infection rate and a stable low virus reproduction rate (R factor), the Federal Ministry of Health recommended on April 28th, 2020, that hospital capacities for elective interventions re-open. The impact of the lockdown on patient care is currently not predictable. However, a significant decrease in hospitalizations in the context of ST-elevation myocardial infarctions (STEMI) has recently been described [4, 5]. Data for patients suffering from tachycardia or bradycardia are lacking.

The aim of this nationwide survey was to analyze the impact of the first lockdown on electrophysiological interventions and patient care. These data might be of interest, since numbers of new infections recently rose again.

## Methods

All centers for invasive electrophysiology and device therapy certified by the German Society for Cardiology (DGK) were contacted. For this purpose, all leading electrophysiologists and employees were primarily contacted. A secondary call was made via social media (Twitter). An online link to the survey was also provided via the newsletter of the DGK Working Group for Electrophysiology (AGEP). The questionnaire comprised 19 questions about invasive treatment volumes for tachycardia and bradycardia before and during the lockdown period. The number of COVID-19 patients treated at these centers and the incidence of cardiac arrhythmia in this patient population were also recorded. All data collected were evaluated anonymously.

## Statistics

Continuous variables were summarized as median ± standard deviation or median (interquartile range; IQR). Categorical variables were expressed as frequencies (percentages).

## Results

### Characteristics of participating centers

**Fig. 1:**
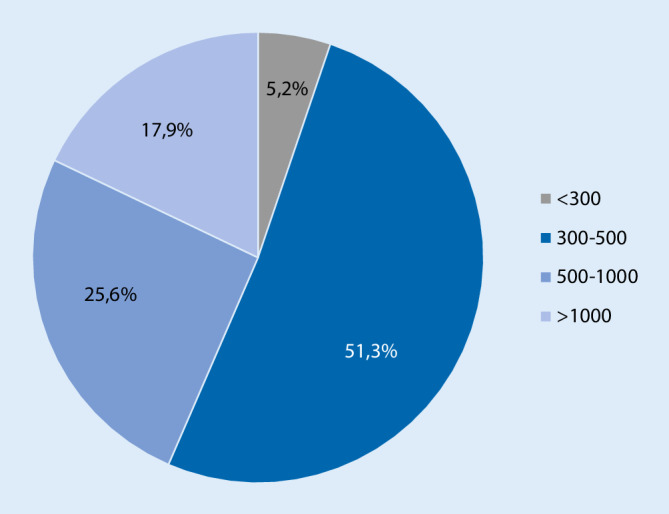
Ablation volumes of the participating centers

A total of 196 sites for invasive electrophysiology certified by the DGK were contacted. The questionnaire was answered in full by 40/196 (20.4%) cardiology centers. These centers cumulatively perform 24,648 ablation procedures/year and thus represent approximately 34% (24,648/72,548) of the estimated German ablation treatments [6]. The vast majority of institutions were academic teaching hospitals (50%) and university hospitals (42.5%), whereas 3/40 (7.5%) were private hospitals (Table 1). With regard to the catheter ablation volume at these centers, 75.6% perform approximately 300–1000 ablations/year and thus represent medium to high volume ablation centers (Fig. 1). The majority of the centers (33/40; 82.5%) were so-called primary COVID-19 hospitals (level-1). A total of 2205 patients with SARS-CoV‑2 infection were hospitalized in these hospitals during the survey period, of which 644/2205 patients (29%) were ventilated invasively. On average, 50 patients (IQR 22; 80) with COVID-19 were treated per center and 15 (IQR 8; 13) of these patients were ventilated invasively (Table 1).

**Table 1:** Characteristics of participating centers

Centers, *n*	40
University hospital, *n* (%)	17/40 (42.5)
Academic teaching hospital, *n* (%)	20/40 (50)
Private hospital, *n* (%)	3/40 (7.5)
Primary COVID-19 (level-1) hospital, *n* (%)	33/40 (82.5)
SARS-CoV-2 patients, *n*	2205
SARS-CoV-2 patients/center	50 (IQR 22; 80)
Ventilated SARS-CoV-2 patients	644/2205 (29)
Ventilated SARS-CoV-2 patients/center	15 (IQR 8; 30)

Metric data are summarized as means +/− standard deviations or as medians [25th and 75th percentiles]. Categorical data are presented as *N* (%). *COVID-19* coronavirus disease 2019, *SARS-CoV‑2* severe acute respiratory syndrome coronavirus 2, *IQR* interquartile range

### Tachycardia and bradycardia in COVID-19 patients

**Fig. 2:**
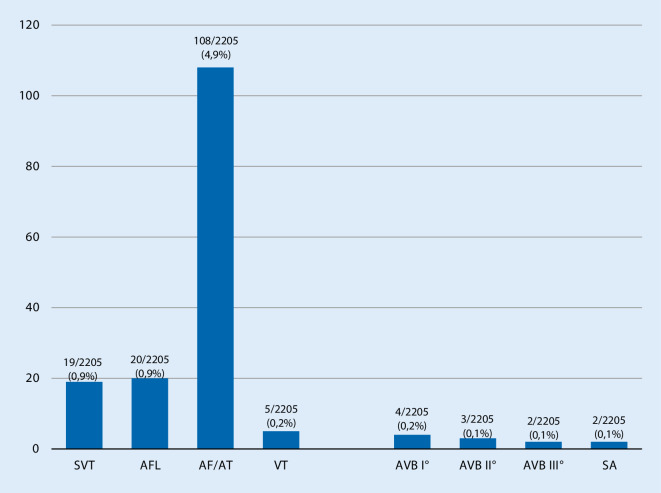
Number of reported cases of tachycardia and bradycardia in severe acute respiratory syndrome coronavirus 2 patients. *SVT* supraventricular tachycardia, *AFL* atrial flutter, *AF* atrial fibrillation, *AT* atrial tachycardia, *VT* ventricular tachycardia, *AVB* atrioventricular block, *SA* sinus arrest

Tachycardia was observed in 152/2205 (6.9%) SARS-CoV-2 patients. The majority of these patients suffered from atrial fibrillation or atrial tachycardia (108/2205; 4.9%). Supraventricular tachycardia (SVT) was documented in 19/2205 (0.9%) SARS-CoV-2 patients, whereas atrial flutter and ventricular tachycardia occurred in 20/2205 (0.9%) and 5/2205 (0.2%) patients, respectively. Electrical cardioversion was performed in 29/2205 (1.3%) patients (see Fig. 2).

Bradycardias were present in 11/2205 (0.5%) of the patients: Sinus arrest occurred in 2/2205 (0.1%), and first-, second-, and third-degree arteriovenous (AV) block in 4/2205 (0.2%), 3/2205 (0.1%), and 2/2205 (0.1%) patients, respectively (see Fig. 2).

### Ablation volume before and during COVID-19 lockdown

Overall, the participating centers stated that they had performed 24,648 ablations per year prior to the COVID-19 pandemic. A cumulative ablation volume of 2054 ablations per month before the COVID-19 period can be assumed. During the lockdown period, there was a decrease to 1218 ablations and thus a reduction of 41% in performed procedures. During the entire lockdown, no patient with SARS-CoV‑2 infection underwent ablation treatment at these centers. Left atrial ablation procedures (pulmonary vein isolation [PVI] or left atrial tachycadia [LAT] ablation) accounted for the majority of these procedures (16,077/24,648; 65.2%). Treatment of these arrhythmias resulted in a reduction in the treatment volume of 1340 ablations/month during the pandemic to 715 ablations/month (47.1%). In the treatment of supraventricular tachycardia (SVT) (6532/24,648; 26.5%) there was a 29% reduction (monthly ablation volume before lockdown: 544 procedures; monthly ablation volume during lockdown: 386). Annually, treatment of ventricular tachycardia (VT) was 8.3% in the whole cohort (2036/24,648 ablations), with a reduction of 31.2% across all centers (monthly ablation volume before lockdown: 170; monthly ablation volume during lockdown: 117), see Table 2 for details.

**Table 2:** Number of catheter ablations/month before and during lockdown period

Procedure	Before lockdown (month), *n*	During lockdown (month), *n*	Reduction (%)
AF/AT	1340	715	47
SVT/AFL	544	386	29
VT	170	117	31
Total	2054	1218	41

*AF* atrial fibrillation, *AT* atrial tachycardia, *SVT* supraventricular tachycardia, *AFL* atrial flutter, *VT* ventricular tachycardia

### Reduction in ablation procedures before and during lockdown

**Fig. 3:**
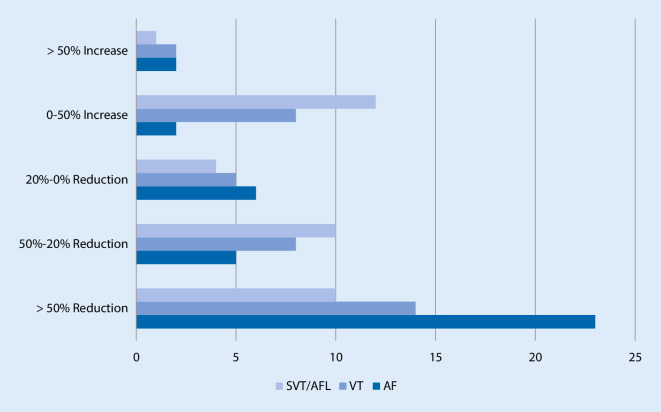
Decrease/increase in ablation volume

The majority of centers (60.5%) reduced their numbers of left atrial interventions by 100–50%. Whereas 13.2% of the centers decreased by 50–20%. However, 10.5% of the centers reported an increase in the number of left atrial interventions performed. With regard to SVT ablations, 27% of centers reduced these by 100–50%, whereas 35% of centers increased their ablation volume. Concerning treatment of ventricular tachycardia, the number of ablation procedures was reduced by 100–50% at 37.8% of centers. Ablation numbers increased compared to the monthly average before the COVID-19 period in 27.1% of centers (see Fig. 3).

### Pacemaker implantations before and during the lockdown

The participating centers stated that they performed a total of 8853 pacemaker implantations per year. The operation volume decreased in all centers: 709 pacemakers were implanted prior to COVID-19 and 579 during the lockdown period. An overall reduction of 18% was observed here. Pacemaker implantation was performed in 4/2205 (0.2%) patients with SARS-CoV‑2 infection.

### Re-hospitalization due to postponed interventions during the lockdown

Due to postponed catheter ablations or pacemaker implantations, 22/40 (55%) centers reported a worsening of clinical symptoms or a requirement for earlier re-hospitalization of their patients.

## Discussion

The aim of this survey was to investigate the direct implications of the COVID-19 pandemic on the patient care situation of rhythmological patients in German electrophysiological centers. As a result of the Federal Government’s request to postpone all non-emergency and elective interventions to free up emergency capacities, the invasive treatment volume was reduced accordingly in cardiological centers.

This survey showed an overall reduction of 41% in catheter ablations and an 18% reduction in pacemaker implantations. The participating centers represent approximately 34% of all German ablation procedures. However, there is no precise information about the exact number of ablations performed in Germany, due to a lack of a centralized quality control in the field of catheter ablation. This needs to be calculated on the basis of voluntary feedback [6].

The majority of centers participating in this survey were medium-sized electrophysiological ablation centers with >300 procedures/year and 82.5% were primary COVID-19 hospitals (level-1). During the lockdown period, these centers treated 2205 COVID-19 patients, of whom 644 patients were ventilated invasively. This may enable a cross-sectional analysis of the impact on patients suffering from cardiac arrhythmia during the lockdown period.

### Tachycardia and bradycardia

The incidence of arrhythmia in SARS-CoV-2 patients is still unknown. The authors believe that the number of cardiac arrhythmias documented in this work are generally somewhat underestimated, since explicitly only rhythmologists and no intensive care physicians were questioned. Also, in clinical practice, rhythmologists may not always be involved in intensive-care-unit patients with stable rhythm disorders. Furthermore, it cannot be clearly determined from this data collection whether the reported rhythm disorders are to be interpreted as epiphenomena or whether they are clearly associated with the underlying infectious disease.

### Electrophysiological procedures before and during lockdown

The majority of arrhythmia cases treated by catheter intervention before and during the lockdown were accounted for by left atrial arrhythmia. As required by policy, a clear reduction in the number of treated elective patients was observed. In 10% of centers, there was an increase in catheter interventions. There may be a number of reasons for this. The fact that some of patients, after postponing their elective appointments, presented to an emergency department at their own center or at another hospital due to a worsening of clinical symptoms may play a role. Another important aspect is that outpatient care for these patients was also not adequately guaranteed during the lockdown period, especially since specialized rhythmological outpatient clinics or referring physicians also reduced their office hours. The consequence of this reduction can also be derived from this survey: 55% of centers report re-hospitalizations or worsening of their patients’ clinical situation due to the postponement of the ablation procedure or device implantation. However, it is not possible to determine with this data set at this point how many of these patients underwent emergency ablation treatment or emergency pacemaker implantation. Catheter ablation of ventricular tachycardia was reduced by 31% in this patient population. A complete reduction in these interventions is usually not possible, since ventricular tachycardia may represent an urgent treatment indication and any additional implantable cardioverter defibrillator shock significantly increases mortality [7]. With regard to SVT, the reduction was comparatively moderate. This could be due to the fact that ablation in SVT is considered to be relatively uncomplicated and associated with a low periprocedural risk of complications and a short hospital stay [8]. Use of ICU capacities and, in particular, invasive ventilation after ablation is generally not to be expected in this patient population. As expected, there was only a slight decrease in pacemaker implantations, as higher-grade AV blocks generally require immediate care.

Finally, the direct implications of the current pandemic and the reduction in cardiological procedures is not fully elucidated. However, there are initial studies from China and Italy that show not only a reduction in coronary intervention, but also a reduction in hospitalizations for STEMI [4]. This was also underlined by a recent survey by the European Cardiology Society [9]. However, the rhythmological effects of the lockdown are currently still unclear and require further investigation, especially since this survey only focused on the elective program. Patients that received an emergency intervention or pacemaker implantation were not noted. However, a recently published analysis showed a reduction in rhythmological emergency visits (13–27%) [10].

In conclusion, the reduction in interventional treatment showed a deterioration in the clinical care situation of rhythmological patients during the lockdown period. Due to the measures implemented in Germany, an initial decline in the number of new infections with SARS-CoV‑2 was observed in the last 2 months. However, a renewed increase in infections with SARS-CoV‑2 has recently been documented in several regions of Germany. This survey provides a first overview suggesting that any renewed reduction of rhythmological procedures should be carried out carefully in order to prevent a risk to this patient population.

## Limitation

As expected, not all EP centers responded to this survey and not all centers can be identified. Importantly, these data rely on self-reporting.

The incidence of arrythmia might be underestimated, since only electrophysiologists were contacted in this survey.

## Conclusion

These results demonstrate a significant decline in elective rhythmological procedures during the lockdown, as required by the German Federal Government. At the same time, however, more than half of the participating centers reported an increase in patient re-hospitalizations due to postponed procedures.
